# Identification and Characterization of Non-Tuberculous Mycobacteria Isolated from Tuberculosis Suspects in Southern-Central China

**DOI:** 10.1371/journal.pone.0114353

**Published:** 2014-12-02

**Authors:** Xiao-li Yu, Lian Lu, Gao-zhan Chen, Zhi-Guo Liu, Hang Lei, Yan-zheng Song, Shu-lin Zhang

**Affiliations:** 1 School of Biology and Pharmaceutical Engineering, Wuhan Polytechnic University, Wuhan, China; 2 Shanghai public health clinical center, Shanghai, China; 3 Department of Immunology and Microbiology, Shanghai Jiao Tong University School of Medicine, Shanghai, China; Beijing Institute of Microbiology and Epidemiology, China

## Abstract

The incidence of non-tuberculous mycobacteria (NTM)-related death has increased globally recently. To obtain information of the species and characterization of pathogens involved in NTM pulmonary infection in Southern-central China, we identified 160 non-tuberculous infection cases from 3995 acid-fast bacilli (AFB)-positive tuberculous suspects. We then randomly selected 101 non-tuberculous patients, isolated bacteria from their sputa and genotyped the pathogens using the 16S rRNA gene and 16S-23S rRNA internal transcribed spacer sequences. *M. intracellulare* (32.67%, 33/101), *M. abscessus* (32.67%, 33/101) and *M. fortuitum* (7.92%, 8/101) are identified in these isolates. Surprisingly, non-mycobacteria including *Gordonia* (8.91%, 9/101), *Nocardia* (5.94%, 6/101) and *Tsukamurella* (0.99%, 1/101) are also discovered, and the case of *Tsukamurella pulmonis* infection is first discovered in Southern-central China. Moreover, species of *M. mucogenicum* group, *M. chubuense*, *M. kansasii*, *M. gastri*, *M. avium*, *M. porcinum* and *M. smegmatis* are identified. In addition, nine immune compromised cases (8.91%, 9/101), including type two diabetes mellitus and HIV/AIDS are found to be infected with non-tuberculous bacteria. This study revealed the distribution and characteristics of non-tuberculous AFB pathogen infection occurred in Southern-central China, and suggested that physicians should be alert of the emerging of NTM and non-mycobacteria infection in AFB positive cases and take caution when choosing chemotherapy for tuberculosis-like pulmonary infections. Generally, this study may help with the development of new strategy for the diagnosis and treatment of mycobacterial infection.

## Introduction

*Mycobacteria* that do not contain *Mycobacterium tuberculosis* (MTB) complex and that do not cause Hansens disease are known as non-tuberculous mycobacteria (NTM). NTM are environmental organisms which opportunistically cause diseases in animals or human[1] and they are increasingly recognized as pathogens in humans [2]. Importantly, most NTM (except *Mycobacterium kansasii*) are inherently resistant to or only partially susceptible to the standard anti-tubercular drugs[3]. Thus, the diagnosis of NTM infection is critical for choosing effective treatment plan.

The most common pulmonary non-tuberculous mycobacterial pathogens are *M. avium*, *M. intracellulare*, *M. chelonae*, *M. abscessus* and *M. fortuitum*, while their species and prevalence vary with the geographical locations [3], [4]. In addition, some weakly acid fast isolates, such as *Gordonia* and *Nocardia*, which are not *Mycobacteria*, are also reported to infect humans[5], [6].

Identification of mycobacteria to the species level by conventional biochemical tests is time consuming, leading to significant delay in diagnosis [7]. Therefore, it is particularly important to accurately and rapidly identify NTM for the correct epidemiological control and specific treatments. 16S rRNA gene analysis is the standard method for identification of mycobacteria. However, limitation of the analysis is evident because some NTM species share the same or very similar 16S rRNA sequence [8], and the basal sequence diversity within the genus *Mycobacterium* is rather low. The polymorphism of 16S-23S rRNA internal transcribed spacer (ITS) sequence in *Mycobacteria* is higher than that of the 16S rRNA gene, so ITS sequence can be used to differentiate the strains of intra-species of *Mycobacteria*. Now ITS is used as ideal target genes in classification and identification of mycobacterial intra-species and ITS sequence serves as an effective supplement for identification of closely related species that 16S rRNA gene sequence can not differentiate[9].

In this study, non-tuberculous acid fast isolates were collected from the sputa of tuberculosis suspects in Southern-central China and were characterized based on the 16S rRNA gene and ITS sequence. The information presented here will increase awareness of Chinese clinicians about NTM.

## Material and Methods

### Ethics statement

Ethical approval is granted by the Ethics Committee of Wuhan Polytechnic University. Sputa from patients with a symptomatic pulmonary infection were obtained from Wuhan Medical Treatment Center in Hubei Province, located in Southern-central China, from July 2011 to July 2013. Informed written consent for the collection of samples and subsequent analysis were provided by all patients.

### Isolation and Culture

Three early morning sputum specimens were collected over three consecutive days from each patient, and then were processed with the standard protocol[10]. After decontamination, each processed sample was cultured onto three Löwenstein-Jensen (L-J) medium at 37°C for 8 weeks. The cultures were inspected weekly and growth was examined by visual inspection for colonies. Positive slides were confirmed by Ziehl-Neelsen staining.

### Phenotype Characterization

AFB were then cultured in p-nitrobenzoic acid (PNB) and thiophene-2-carboxylic acid hydrazide (TCH) modified L-J medium [11]. Isolates that could grow in both PNB and TCH modified L-J medium were considered as NTM, which were subsequently genotyped by 16S rRNA gene and ITS sequencing.

### Molecular identification

The genomic DNA of the bacteria was extracted using classical phenol-chloroform method and stored at −20°C. A set of primers were designed based on the *M. tuberculosis* H37Rv genome sequence[12] using Premier 5.0 (PREMIER Biosoft, Palo Alto, CA, USA) and Oligo 6 software (Molecular Biology Insights, Inc., Cascade, CO, USA). 16S rRNA genes of all isolates were amplified with 16S-F (5′-AGAGTTTGATCCTGGCTCAG-3′) and 16S-R (5′-ACGGGCGGTGTCTACAA-3′) targeting positions 11–1399 of the 16S rRNA gene. ITS-F (16SrRNA) (5′- GTGGGATCGGCGATTGGGAC-3′) positions 1280 to 1299 of the 16S rRNA gene and ITS-R (23SrRNA) (5′- CCACCATGCGCCCTTAGACAC -3′) positions 7 to 27 of the 23S rRNA gene were used to amplify ITS regions. The amplification was done with a 50 µl PCR reaction containing a final concentration of 1 µlM specific primers, 1× PCR Buffer, 1.5 mM MgCl_2_, 2.5 U Taq polymerase (Takara), 200 mM of each deoxynucleoside triphosphate (Takara), 5µl purified DNA. PCR condition for 16S rRNA gene and ITS was: 94°C for 5 minutes, 38 cycles of 94°C for 30 seconds, 55°C for 30 seconds and 72°C for 1 minute. A final extension phase of 72°C for 10 minutes was used. PCR products were then separated by electrophoresis using a 1% TBE agarose gel and the amplicons were purified with the E.Z.N.A. MicroElute Cycle-Pure Kit (Omega, Lilburn, GA, USA).

Direct sequencing was performed on PCR products with DNA Analyzer (3730×l, ABI). Primers 16S-F, 16S-R, ITS-F were used for sequencing. 16S rRNA gene was sequenced in both directions while ITS sequences were sequenced in single direction. Sequence data were assembled and analyzed by CLUSTAL W software (European Bioinformatics Institute). Fragment sizes are shown in Table 1. Molecular typing and species identification were performed using BLAST search (GenBank database sequences). Unequivocal identification was defined as 99% or 100% sequence homology with a unique species sequence in GenBank.

**Table 1:** Molecular identification by aligning 16S rRNA gene and ITS sequences (n =  101).

Genus	16S rRNA gene			ITS sequence		
	Species	Strains	Fragment size (bp)	Species	Strains	Fragment size (bp)
***Mycobacterium***	MAC	36(35.64%)	1329–339	*M. intracellulare*	33(32.67%)	352–374
				*M. avium*	3(2.97%)	341–509
	*M. chelonae -abscessus* Complex	33(32.67%)	1304–1324	*M. abscessus*	33(32.67%)	367–572
	*M. fortuitum* group	11(10.89%)	1317–1329	*M. porcinum*	3(2.97%)	454–610
				*M. fortuitum*	8(7.92%)	422–590
	*M. kansasii-gastri* Complex	2(1.98%)	1331	*M. kansasii*	2(1.98%)	344
	*M. smegmatis group*	1(0.99%)	1323	*M. smegmatis*	1(0.99%)	620
	*M. mucogenicum group*	1(0.99%)	1323	*M. sp.*	2(1.98%)	633–658
	*M. chubuense*	1(0.99%)	1316			
***Gordonia***	*G. bronchialis*	6(5.94%)	1319–1325	*G. ronchialis*	6(5.94%)	604–647
	*G. paraffinivorans*	3(2.97%)	1322–1334	*G. sp.*	3(2.97%)	572–698
***Nocardia***	*N. farcinica*	4(3.96%)	1314–1320	*N. farcinica*	4(3.96%)	379–405
	*N. puris*	2(1.98%)	1319–1320	*N. sp.*	2(1.98%)	554–555
***Tsukamurella***	*Tsukamurella sp.*	1(0.99%)	1318	*T. pulmonis*	1(0.99%)	661

## Results

### Phenotype Characterization

From 3995 acid fast isolates, 160 are identified as NTM, based on their ability to grow in medium containing PNB and TCH. Double/mixed infections (MTB+ NTM/Other AFB) are not detected in this patient cohort. We randomly selected 101 NTM isolates for further characterization.

Patient characteristics of the 101 suspected tuberculosis cases are shown in Table 2. All of them are hospitalized, treated and discharged except that one patient died. Sixty nine patients (68.32%) are male and forty-six (45.54%) are 46–65 years old. Over half of them (58.42%) had a history of TB treatment. HIV infection is found in one (0.99%) patient. Eight cases of type two diabetes mellitus (T2DM) are found. Sixty individuals (59.41%) had respiratory system symptoms.

**Table 2:** Characteristics for suspected tuberculosis patients (n = 101).

Characteristic	Patient (%)
**Sex**	
**Male**	69 (68.32%)
**Female**	32 (31.68%)
**Age**	
**<20**	1 (0.99%)
**21–45**	28 (27.72%)
**46–65**	46 (45.54%)
**>65**	26 (25.74%)
**Previous TB treatment**	59 (58.42%)
**HIV infection**	1 (0.99%)
**Type two diabetes mellitus**	8 (7.92%)
**Any respiratory symptom**	60 (59.41%)

### Molecular identification

Based on 16S rRNA gene analysis (Table 1), we found the most common NTM pathogens are *M. avium-intracellulare* complex (MAC), followed by *M. chelonae-abscessus* complex (including *M. abscessus, M. chelonae, M. bolletii, M. massiliense, M. immunogenum* and *M. franklinii* that share 99% homologies in 16S rRNA gene) and *M. fortuitum* group (including *M. fortuitum*, *M. porcinum*, *M. senegalense*, *M. houstonense*, *M. conceptionense*, *M. boenickei*, *M. setense*, *M. peregrinum, M. septicum*, *M. neworleansense and M. farcinogenes* that share 99% homologies in 16S rRNA gene). In addition, *M. kansasii-gastri* complex, *M. smegmatis* group (including *M. smegmatis* and *M. goodii* which share 99% homologies in 16S rRNA gene), *M. mucogenicum* group (including *M. mucogenicum*, *M. aubagnense* and *M. phocaicum* which share 99% homologies in 16S rRNA gene) and *M. chubuense* are identified. To our surprise, non-mycobacteria pathogens (15.84%, 16/101) such as *Gordonia*, *Nocardia* and *Tsukamurella* are found in this study. *Gordonia*, including *Gordonia bronchialis* and *G. paraffinivorans*, accounted for 8.91% (9/101) and *Nocardia*, such as *Nocardia farcinica* and *N. puris*, accounted for 5.94% (6/101). One isolate of *Tsukamurella sp.* is found in this study.

ITS sequences analysis is used to confirm the results of 16S rRNA gene sequencing. Moreover, ITS sequences analysis gave more definitive identification and classification of NTM. *M. avium*, *M. intracellulare*, *M. abscessus*, *M. fortuitum*, *M. porcinum*, *M. kansasii*, *M. smegmatis* are clearly identified and differentiated from each other. In addition, by using ITS analysis, we are able to further classify the pathogen that accounted for the case of *Tsukamurella* infection as *Tsukamurella pulmonis*. In this study, we are unable to use ITS sequence to differentially identify *M. mucogenicum* group, *M. chubuense*, *G. paraffinivorans* because the ITS sequences of these particular species are unavailable on the public data base that we searched.

## Discussion

Distinguishing NTM from MTB infection is a major challenge in clinic, which requires rapid and sensitive identification of the pathogens [13]. Clinic symptoms are often very similar between NTM and MTB infection which seriously hampered the diagnosis and treatment of MTB and NTM caused diseases [14] since management of mycobacterial infection is species specific [15]. Therefore, rapid detection and identification of the infecting mycobacterial species are desirable for specific chemotherapy and better patient management. Traditional method such as PNB/TCH culture takes several weeks to perform and it can only be used to distinguish NTM from MTB while it can not be used to categorize NTM. In recent years, the gene sequencing techniques have been successfully employed for rapid species classification. 16S rRNA gene and ITS sequence serve as complementary methods for species genotyping [16]. In the current study, by using the sequencing techniques, we successfully genotyped the 101 randomly selected non-tuberculous isolates to species level.

In this study, we found MAC is the main NTM pathogens in Southern-central China, consistent with the results of previous report in India, Korea and Guangxi province of China[3], [17]. The second common NTM pathogen is *M. abscessus* (32.67%, 33/101), similar to the findings of a study in Taiwan[18]. The third is *M. fortuitum* (7.92%, 8/101), in accordance with studies performed in Shangdong province and Taiwan[18], [19], while a study in Shanghai showed that *M. fortuitum* is second commonest isolate [20]. Generally, the distribution of NTM species may vary with geographic region.

Unexpectedly, a relatively high incidence of non-mycobacteria including *Gordonia* (8.91%, 9/101), *Nocardia* (5.94%, 6/101) and *Tsukamurella* (0.99%, 1/101) is discovered. Interestingly, the occurrence of *Gordonia* is similar to a report in Taiwan [21]. There are six isolates of *Nocardia*, which often cause chronic lung diseases, as reported in the United States and Taiwan [22], [23]. In addition, we first reported a *T. pulmonis* infection case in Southern-central China while it has previously been reported in Hong Kong and Taiwan [24], [25].

Particularly, nine cases (8.91%, 9/101) with immunodeficiency symptoms, including T2DM and HIV/AIDS are discovered in this study. Six T2DM patients are found to be infected with non-mycobacteria (two strains of *G. paraffinivorans*, one of *G. bronchialis*, *N. puris, N. farcinica* and *T. pulmonis*). The other two T2DM patients are infected with *M. intracellulare* and the one HIV-infected patient is accompanied with *M. abscessus* infection. The identification of these pathogens in T2DM and AIDS suggests that non-tuberculous AFB opportunistic infections should be taken into consideration in immune-compromised patients.

Clinical laboratory and image findings are similar in patients with drug resistant TB and NTM infections, or non-mycobacterial AFB infections. The majority of these patients present with positive smear for AFB and they usually have no response to first-line and some second-line anti-TB chemotherapy. Physicians should be aware of the emerging of NTM and non-mycobacteria infection in AFB positive cases so they may choose appropriate chemotherapy for pulmonary infections. This study may help develop new strategy for the diagnosis of tuberculosis-like infection, which can lead to effective treatment of the diseases.

## References

[1] Wallace R (2010) History of MAC. Available: http://www.maclungdisease.org/history-of-mac. Accessed 06 September 2014.

[2] Simons S, van Ingen J, Hsueh PR, Van Hung N, Dekhuijzen PN, et al (2011) Nontuberculous mycobacteria in respiratory tract infections, eastern Asia. Emerg Infect Dis 17:343–349. 21392422 10.3201/eid1703100604 PMC3165997

[3] Gopinath K, Singh S (2010) Non-tuberculous mycobacteria in TB-endemic countries: are we neglecting the danger? Plos Neglect Trop D 4:e615. 10.1371/journal.pntd.0000615 PMC2860495 20436962

[4] Brown-Elliott BA, Wallace RJ (2002) Clinical and taxonomic status of pathogenic nonpigmented or late-pigmenting rapidly growing mycobacteria. Clin Microbiol Rev 15:716–746. 12364376 10.1128/CMR.15.4.716-746.2002 PMC126856

[5] Chen Y, Tong C, Jiang Y, Shui Y, Tian L, et al (2010) A case of lung infection caused by Gordonia bronchialis. Microbes Infect 5:93–99.

[6] Tachezy M, Simon P, Ilchmann C, Vashist YK, Izbicki JR, et al (2009) Abscess of adrenal gland caused by disseminated subacute Nocardia farcinica pneumonia. A case report and mini-review of the literature. BMC Infect Dis 9:194. 19954528 10.1186/1471-2334-9-194 PMC2792227

[7] Springer B, Stockman L, Teschner K, Roberts GD, Bottger EC (1996) Two-laboratory collaborative study on identification of mycobacteria: molecular versus phenotypic methods. J Clin Microbiol 34:296–303. 8789004 10.1128/jcm.34.2.296-303.1996 PMC228786

[8] Simmon KE, Brown-Elliott BA, Ridge PG, Durtschi JD, Mann LB, et al (2011) Mycobacterium chelonae-abscessus complex associated with sinopulmonary disease, Northeastern USA. Emerg Infect Dis 17:1692–1700. 21888796 10.3201/eid1709.101667 PMC3322061

[9] Zhang SL, Shen JG, Shen GH, Sun ZQ, Xu PH, et al (2007) Use of a novel multiplex probe array for rapid identification of Mycobacterium species from clinical isolates. World J Microb Biot 23:1779–1788. 10.1007/s11274-007-9428-1 27517834

[10] Kent PT, Kubica GP (1985) Public health mycobacteriology: a guide for the level III laboratory. US Department of Health and Human Services, Centers for Disease Control and Prevention, Atlanta, USA.

[11] Yu XL, Wen ZL, Chen GZ, Li R, Ding BB, et al (2014) Molecular characterization of multidrug-resistant Mycobacterium tuberculosis isolated from South-central in China. J Antibiot (Tokyo) 67:291–297. 24326341 10.1038/ja.2013.133

[12] Camus JC, Pryor MJ, Médigue C, Cole ST (2002) Re-annotation of the genome sequence of Mycobacterium tuberculosis H37Rv. Microbiology+ 148:2967–2973. 12368430 10.1099/00221287-148-10-2967

[13] Gopinath K, Singh S (2009) Multiplex PCR assay for simultaneous detection and differentiation of Mycobacterium tuberculosis, Mycobacterium avium complexes and other Mycobacterial species directly from clinical specimens. J Appl Microbiol 107:425–435. 19302308 10.1111/j.1365-2672.2009.04218.x

[14] McGrath EE, Anderson PB (2010) The therapeutic approach to non-tuberculous mycobacterial infection of the lung. Pulm Pharmacol Ther 23:389–396. 20542128 10.1016/j.pupt.2010.06.002

[15] Montessori V, Phillips P, Montaner J, Haley L, Craib K, et al (1996) Species distribution in human immunodeficiency virus-related mycobacterial infections: implications for selection of initial treatment. Clin Infect Dis 22:989–992. 8783698 10.1093/clinids/22.6.989

[16] Roth A, Fischer M, Hamid ME, Michalke S, Ludwig W, et al (1998) Differentiation of phylogenetically related slowly growing mycobacteria based on 16S-23S rRNA gene internal transcribed spacer sequences. J Clin Microbiol 36:139–147. 9431937 10.1128/jcm.36.1.139-147.1998 PMC124824

[17] Park Y, Lee C, Lee S, Yang S, Yoo C, et al (2010) Rapid increase of non-tuberculous mycobacterial lung diseases at a tertiary referral hospital in South Korea [Short communication]. Int J Tuberc Lung D 14:1069–1071. 20626955

[18] Lai C-C, Tan C-K, Chou C-H, Hsu H-L, Liao C-H, et al (2010) Increasing incidence of nontuberculous mycobacteria, Taiwan, 2000–2008. Emerg Infect Dis 16:294. 20113563 10.3201/eid1602.090675 PMC2958002

[19] Jing H, Wang H, Wang Y, Deng Y, Li X, et al (2012) Prevalence of nontuberculous mycobacteria infection, China, 2004–2009. Emerg Infect Dis 18:527. 22376989 10.3201/eid1803.110175 PMC3309567

[20] Wang HX, Yue J, Han M, Yang JH, Gao RL, et al (2010) Nontuberculous mycobacteria: susceptibility pattern and prevalence rate in Shanghai from 2005 to 2008. Chin Med J (Engl) 123:184–187. 20137367

[21] Lai C, Wang C, Liu C, Tan C, Lin S, et al (2010) Infections caused by Gordonia species at a medical centre in Taiwan, 1997 to 2008. Clin Microbiol Infec 16:1448–1453. 19832703 10.1111/j.1469-0691.2009.03085.x

[22] Tan CK, Lai CC, Lin SH, Liao CH, Chou CH, et al (2010) Clinical and microbiological characteristics of nocardiosis including those caused by emerging Nocardia species in Taiwan, 1998–2008. Clin Microbiol Infec 16:966–972. 19860823 10.1111/j.1469-0691.2009.02950.x

[23] Brown-Elliott BA, Brown JM, Conville PS, Wallace RJ Jr (2006) Clinical and laboratory features of the Nocardia spp. based on current molecular taxonomy. Clin Microbiol Rev 19:259–282. 16614249 10.1128/CMR.19.2.259-282.2006 PMC1471991

[24] Woo PC, Fong AH, Ngan AH, Tam DM, Teng JL, et al (2009) First report of Tsukamurella keratitis: association between T. tyrosinosolvens and T. pulmonis and ophthalmologic infections. J Clin Microbiol 47:1953–1956. 19369436 10.1128/JCM.00424-09 PMC2691093

[25] Liu CY, Lai CC, Lee MR, Lee YC, Huang YT, et al (2011) Clinical characteristics of infections caused by *Tsukamurella* spp. and antimicrobial susceptibilities of the isolates. Int J Antimicrob Ag 38:534–537. 10.1016/j.ijantimicag.2011.07.018 22014886

